# Persistent brainstem abscess requiring repeat microsurgical drainage: case report

**DOI:** 10.1093/jscr/rjab376

**Published:** 2021-08-31

**Authors:** Wesley Shoap, Ellery A Hayden, George A Crabill

**Affiliations:** Department of Neurosurgery, Louisiana State University, New Orleans, LA, USA; Department of Neurosurgery, Louisiana State University, New Orleans, LA, USA; Department of Neurosurgery, Louisiana State University, New Orleans, LA, USA

**Keywords:** brainstem, abscess, neurosurgery

## Abstract

In this paper, we present a patient who was treated for a pontine abscess at our institution. This patient underwent sub-occipital craniotomy for microscopic abscess drainage after which cultures grew *Streptococcus intermedius*. She was treated with antibiotics but failed to show clinical improvement and was taken back to the operating room for repeat abscess drainage. Clinical improvement was seen after the second operation. This case report describes open surgical technique as a safe and effective way of treating brainstem abscess.

## INTRODUCTION

Solitary brainstem abscesses are extremely rare and account for <2% of all brain abscesses [1]. The incidence of brainstem abscess comprises up to 8% of intracranial space occupying lesions in developing countries and up to 2% in developed countries [2]. Brainstem abscesses in the literature are most often pontine or mesencephalic in both pediatric and adult populations [3–5]. Adult data suggest a 2.4:1 and 15:9 male predominance, while studies in pediatric populations show a slight 15 to 9 predominance in women [1, 3]. According to meta-analysis data, mean age of onset of is 34–37 years old [1, 4].

The most common causative organisms include *Streptococcus* and *Staphylococcus* species, although many others exist [6–8]. Most often reported risk factors include oral or otalaryngologic infection that predisposes to contiguous spread, thoracic or abdominal infection, immunosuppression, surgery, trauma and bacteremia [7]. Of these, oral/otalaryngologic infection and immunosuppression significantly contribute to the risk of developing brainstem abscess, specifically.

Treatments include antibiotics with or without stereotactic aspiration or open microsurgical drainage [4]. While there is some debate surrounding complete excision versus repeated aspirations in treating brain abscesses, current recommendations suggest that 4–8 weeks of high-dose antibiotics with or without repeat stereotactic aspirations pose less risk to essential brain tissue than complete excision.

## CASE ILLUSTRATION

**Figure 1:**
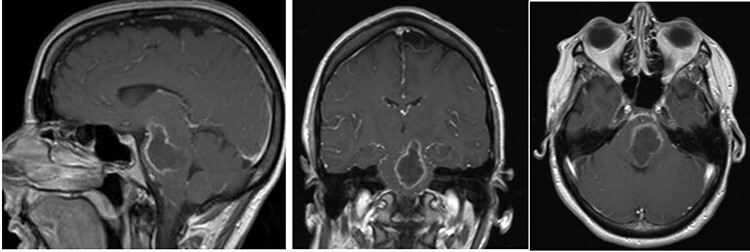
Pe-op axial, coronal and sagittal T1 post-contrast MRIs demonstrating rim-enhancing pontine abscess.

A 44-year-old female presented initially to an outside hospital with difficulty in speaking, right-sided facial droop and difficulty in walking. She also endorsed a 1-week history of intermittent headache. Magnetic resonance imaging (MRI) of brain demonstrated a ring-enhancing pontine lesion with surrounding vasogenic edema consistent with a brainstem abscess measuring 3.6 × 2.4 × 3.7 cm (Fig. 1a–c). There were no areas of ischemia. Pertinent history revealed no dental, cardiac or pulmonary source of infection. She had no history of intravenous drug use.

**Figure 2:**
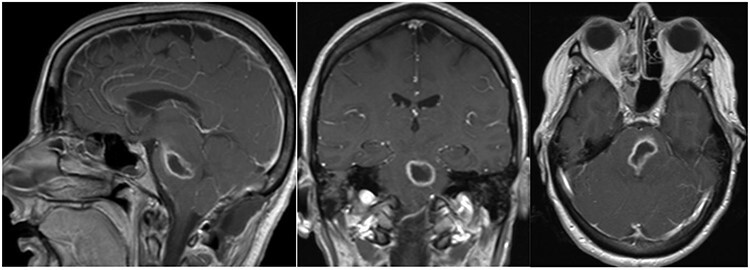
Post-op axial, coronal and sagittal T1 post-contrast MRIs demonstrating decrease size of rim enhancing pontine abscess.

On neurologic exam, she exhibited dysarthric speech with intact cognition and appropriate mental status. Pupils were 4 mm and were equal, round and reactive to light. She had nystagmus on right gaze and had a left gaze palsy. Face sensation was diminished to light touch on the right and she exhibited right facial weakness as well. Hearing was intact and palate elevated to the midline. Tongue had normal motion. She exhibited significantly increased tone with contracture in the right upper extremity. Strength was 4/5 in the right lower extremity, but sensation to light touch was intact throughout her extremities and trunk. Deep tendon reflexes were 3+ on bilateral upper extremities and 2+ on bilateral lower extremities.

C-reactive protein (CRP) was 1.4 mg/dl and erythrocyte sedimentation rate (ESR) was 28 mm/h. Cerebrospinal fluid (CSF) studies were relatively benign. Cryptococcal antigen was negative and spinal fluid exhibited no xanthochromia. She was started on vancomycin, ceftriaxone and metronidazole for probable central nervous system abscess and was transferred to our facility for neurosurgical intervention.

Three days after initial presentation, she underwent sub-occipital craniotomy for microsurgical abscess drainage**.** Initial consideration was given to a stereotactic procedure; however, an open approach was ultimately chosen due to surgeon preference. Intraoperative neuro navigation was used with subsequent creation of a posterior fossa craniotomy. This approach was chosen over a retrosigmoid approach, given the midline nature of the lesion. The dura was opened over the right cerebral hemisphere and tonsil at which time pus was expressed and was cultured immediately at the operative site. We were unable to dissect the roof of the fourth ventricle secondary to a large volume of pus and instead retracted the bilateral tonsils and resected a small amount of the inferior vermis. Facial colliculus was localized and an incision was made in the midline medial raphae. Incision into the brainstem did not express any purulence, so the wound was copiously irrigated and subsequently closed without advancing further into the brainstem. Cultures from the abscess grew step intermedius and antibiotics were narrowed accordingly. Over the next several weeks, the patient remained afebrile with no leukocytosis, but there was concern for persistent infection due to lack of clinical improvement. Repeat MRI demonstrated enlargement of the residual abscess (Fig. 2a–c), and she was taken back to the operating room for repeat abscess drainage via the same sub-occipital craniotomy incision. During this procedure, the roof of the fourth ventricle was visualized and dissected through before localization of the vagal trigone. Midline raphae was once again opened and intraoperative MRI navigation confirmed the locations of the abscess. Microscopic dissection was performed in the midline between facial colliculi, and spinal needle was passed into the opening with subsequent aspiration of 9-ml purulent fluid, re-aspiration attempt and closure. Repeat gram stain and culture failed to reveal any organisms.

**Figure 3:**
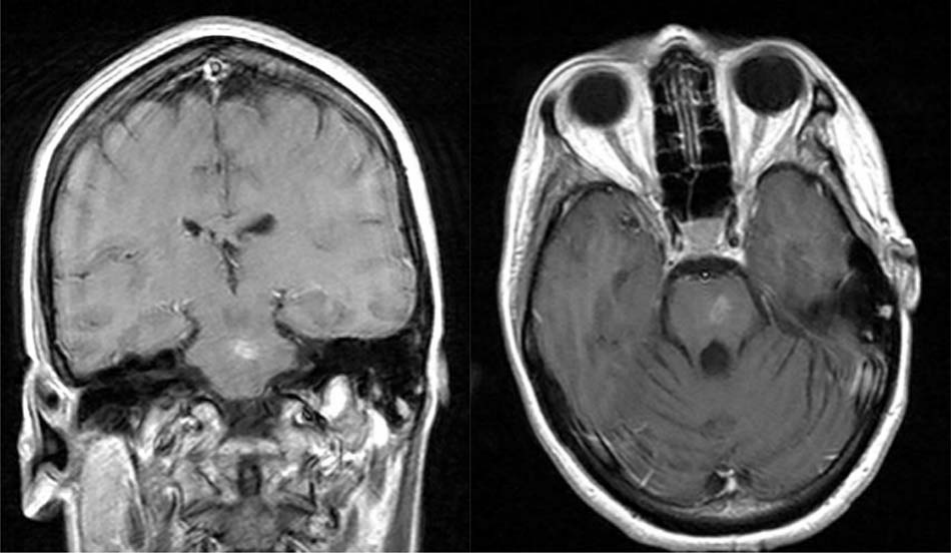
Post-op axial and coronal MRIs, 11 weeks following discharge, demonstrating resolution of the pontine abscess with small area of residual scarring; source: http://pacsweb.wjmc.org.

She presented to clinic for follow-up evaluation 11 weeks after discharge. Overall, she is doing very well and ambulating independently with a cane. On physical exam, CNII-XII are intact. She has no bulbar deficits. She has 4/5 strength in all major muscle groups on the right side. Repeat MRI has shown near resolution of the abscess (Fig. 3a and b).

## DISCUSSION

Brain abscess is an uncommon condition occurring in 0.76 per 100 000 person years, although it has been increasing over the past four decades. It has been estimated that brainstem abscesses account for 0.5–6% of all intracranial abscesses, signifying an extremely rare condition [4, 9]. In the predominant number of cases, a local source of infection, namely oral or otalaryngologic, is identified [7]. Notably, this patient never had an identifiable source, a phenomena which has been described in a minority of cases. CRP, a maker commonly used to trend response to therapy, was only slightly elevated pre-operatively. Post-operatively, it increased to as high as 22.8 mg/dl. CRP can be used as a reliable surrogate for response to therapy, although in this case, we did not obtain enough data points to create a reliable tend.

A literature search revealed only 31 case reports of brainstem abscesses successfully treated by surgical drainage or stereotactic aspiration from 1984 to 2020. A literature review by Stein *et al*. of surgically treated brainstem abscesses reported from 1984 to 2009 demonstrated that 54% were by stereotactic aspiration and 46% were by an open surgical technique [10]. This case demonstrates that microsurgical drainage of brainstem abscesses is a reasonable and safe option to be considered in the management of brainstem abscesses.

## CONFLICT OF INTEREST STATEMENT

None declared.

## FUNDING

None.
